# Analysis of circulating angiopoietin-like protein 3 and genetic variants in lipid metabolism and liver health: the DiOGenes study

**DOI:** 10.1186/s12263-018-0597-3

**Published:** 2018-04-02

**Authors:** Anne Lundby Hess, Jérôme Carayol, Trine Blædel, Jörg Hager, Alessandro Di Cara, Arne Astrup, Wim H. M. Saris, Lesli Hingstrup Larsen, Armand Valsesia

**Affiliations:** 1 The Department of Nutrition, Exercise and Sports, Faculty of Science, University of Copenhagen, Rolighedsvej 26, 1958 Frederiksberg C, Denmark; 2 Nestlé Institute of Health Sciences, Lausanne, Switzerland; 3 Precision for Medicine, Geneva, Switzerland; 4 The Department of Human Biology, NUTRIM School for Nutrition, Toxicology and Metabolism, Maastricht University Medical Centre, Maastricht, Netherlands

**Keywords:** Angiopoietin-like protein 3, Liver markers, Liver steatosis, Lipid metabolism, Lipoprotein lipase, Protein quantitative trait locus, Single nucleotide polymorphisms

## Abstract

**Background:**

Angiopoietin-like protein 3 (ANGPTL3), a liver-derived protein, plays an important role in the lipid and lipoprotein metabolism. Using data available from the DiOGenes study, we assessed the link with clinical improvements (weight, plasma lipid, and insulin levels) and changes in liver markers, alanine aminotransferase, aspartate aminotransferase (AST), adiponectin, fetuin A and B, and cytokeratin 18 (CK-18), upon low-calorie diet (LCD) intervention. We also examined the role of genetic variation in determining the level of circulating ANGPTL3 and the relation between the identified genetic markers and markers of hepatic steatosis.

**Methods:**

DiOGenes is a multicenter, controlled dietary intervention where obese participants followed an 8-week LCD (800 kcal/day, using a meal replacement product). Plasma ANGPTL3 and liver markers were measured using the SomaLogic (Boulder, CO) platform. Protein quantitative trait locus (pQTL) analyses assessed the link between more than four million common variants and the level of circulating ANGPTL3 at baseline and changes in levels during the LCD intervention.

**Results:**

Changes in ANGPTL3 during weight loss showed only marginal association with changes in triglycerides (nominal *p* = 0.02) and insulin (*p* = 0.04); these results did not remain significant after correcting for multiple testing. However, significant association (after multiple-testing correction) were observed between changes in ANGPTL3 and AST during weight loss (*p* = 0.004) and between ANGPTL3 and CK-18 (baseline *p* = 1.03 × 10^−7^, during weight loss *p* = 1.47 × 10^−13^). Our pQTL study identified two loci significantly associated with changes in ANGPTL3. One of these loci (the *APOA4*-*APOA5-ZNF259*-*BUD13* gene cluster) also displayed significant association with changes in CK-18 levels during weight loss (*p* = 0.007).

**Conclusion:**

We clarify the link between circulating levels of ANGPTL3 and specific markers of liver function. We demonstrate that changes in ANGPLT3 and CK-18 during LCD are under genetic control from *trans*-acting variants. Our results suggest an extended function of ANGPTL3 in the inflammatory state of liver steatosis and toward liver metabolic processes.

**Electronic supplementary material:**

The online version of this article (10.1186/s12263-018-0597-3) contains supplementary material, which is available to authorized users.

## Background

The metabolic syndrome is a cluster of risk factors that increases the risk of diseases such as type 2 diabetes, hypertension, hyperlipidemia, and non-alcoholic fatty liver disease. The prevalence of the metabolic syndrome increases due to a parallel rise in the occurrence of obesity and insulin resistance [1]. This highlights the need for a more detailed understanding of the underlying molecular mechanisms.

One of the key components in the etiology of the metabolic syndrome is dyslipidemia. Angiopoietin-like proteins (ANGPTLs) have been reported to be involved in the regulation of lipid metabolism [2]. The human gene of angiopoietin-like protein 3 (ANGPTL3) is located on chromosome 1 and encodes one of several structurally similar secreted glycoproteins in the ANGPTL family. The ANGPTLs consists of a signal sequence at the N-terminal followed by an α-helical region forming coiled coil domains, and a fibrinogen-like domain at the C-terminal. ANGPTL8 differs in structure, as it lacks a C-terminal fibrinogen-like domain. ANGPTL3 is found in plasma both as a native protein and in cleaved form [2, 3]. The coiled coil domains at the N-terminal decrease the hydrolysis of plasma triglyceride (TG) through inhibition of lipoprotein lipase (LPL) activity and thereby affect the lipid and lipoprotein metabolism [4]. ANGPTL3 is predominantly expressed in the liver and is secreted by the liver both in mice and in humans [5, 6]. ANGPTL3 deficiency results in a dramatic reduction of the plasma concentration of TG and cholesterol [5, 7], and loss of function mutations in *ANGPTL3* are the cause of a recessive form of familial combined hyperlipidemia [8].

In addition to stimulation of lipolysis, ANGPTL3 may be a determining factor in increasing hepatic lipid storage and affecting free fatty acid (FFA)-induced insulin resistance. One study reported a positive association between circulating ANGPTL3 and non-alcoholic steatohepatitis (NASH) [9]. Altogether, ANGPTL3 may be involved in the pathogenesis of the metabolic syndrome and increase the risk of hepatic steatosis.

This study examines the role of ANGPTL3 in lipid metabolism and liver health in the DiOGenes (Diet, Obesity and Genes) study. The DiOGenes study was a randomized, controlled dietary intervention that showed that a reduction in the glycemic index (GI) and an increase in dietary protein content led to an improvement in weight maintenance after an 8-week low-calorie diet (LCD) weight loss in adults [10]. In this study, we first analyze ANGPTL3 concentration in relation to body mass index (BMI), lipid profile, and markers of hepatic steatosis before and during weight loss. Afterwards, we identified genetic variants determining variations of circulating ANGPTL3 level through protein quantitative trait locus (pQTL) analysis and tested their association to ANGPTL3-related covariates.

## Methods

### Study design

The DiOGenes study (registered at http://www.clinicaltrials.gov, NCT00390637) was an intervention study carried out in eight European centers (Bulgaria, the Czech Republic, Denmark, Germany, Greece, the Netherlands, Spain, and the UK). The primary purpose was to examine the effects of dietary protein and GI on weight regain and metabolic and cardiovascular risk factors in overweight and obese families [10–12]. The study included families with at least one overweight or obese parent less than 65 years of age. The participants aimed to lose ≥ 8% of their initial body weight during 8 weeks of a LCD (800 kcal/day with additional use of 200 g of vegetables/day). Subjects achieving ≥ 8% weight loss were included in a 6-month weight maintenance period. Here, the participants were randomized to one of four ad libitum diets differing in GI and dietary protein content or a control diet following the national dietary guidelines in each of the countries [11].

### Ethics

The study was approved by the different local ethical committees. Written informed consent was obtained from all participants, and the study was performed in accordance with the Declaration of Helsinki.

### Clinical measurements

In the study, height was measured at the initial screening visit. Body weight was measured on all of the clinical investigation days together with fasting blood sampling. Total cholesterol, high-density lipoprotein cholesterol (HDL-C), TG, fasting glucose, and insulin were analyzed at the Research Laboratory, Department of Clinical Biochemistry, Gentofte University Hospital, Denmark. Low-density lipoprotein cholesterol (LDL-C) was calculated according to Friedewald’s equation [13].

### Proteomics analyses

Plasma concentrations of ANGPTL3, alanine aminotransferase (ALT), aspartate aminotransferase (AST), adiponectin, fetuin A, fetuin B, and cytokeratin 18 (CK-18) were quantified before and after the LCD intervention using a multiplexed aptamer-based proteomic technology developed by SomaLogic Inc. (Boulder, CO) and measured as relative fluorescence units (RFU) [14, 15]. Data was normalized and calibrated by SomaLogic™ according to standard operating procedures [16]. This was done to remove systematic biases and correct plate-to-plate variation. Additional post-processing steps removed subjects with potential cell lyses as indicated with high hemoglobin levels (> 9 × 10^5^ RFU) and outliers as detected with principal component analyses. Proteins were also checked for outliers and proportion of missing values before log transformation for analysis [17]. Data were available for 1129 proteins in 512 DiOGenes participants. Protein change during the weight loss intervention was computed as the log_2_ fold change between the end and the beginning of the intervention.

### Genotyping

DNA was extracted from EDTA blood buffy coats with a salting out method. The DNA samples were quality checked, quantified, and normalized to approximately 100 ng/ml and 2.0 mg before genotyping. Genotyping was done using Illumina 660 W-quad according to manufacturer’s protocols (Illumina, San Diego, CA). Detailed information about this dataset can be found in Carayol et al. [17]. Briefly, 498,233 single nucleotide polymorphisms (SNPs) were genotyped; after quality check, additional SNPs were imputed using the Michigan Imputation Server [18] and the European 1000 Genomes set reference panel. SNP information was mapped onto NCBI version 37. Information was available for 4,020,654 SNPs in 494 participants with proteomics data.

### QTL mapping

A complete description of the QTL mapping is available in Carayol et al. [17]. In summary, association between SNPs and circulating ANGPTL3 was tested at baseline and during weight loss using linear mixed effect models as implemented in GCTA software adjusting for baseline BMI or change in BMI, center, age, and gender as fixed, and a genetic relationship matrices as random effect [19]. In order to handle the multiple comparisons, *p* values were corrected using SLIDE (Sliding-window method for Locally Inter-correlated markers with asymptotic Distribution Errors corrected), a method based on a multivariate normal distribution similar to classical permutation but much faster [20]. Considering the large number of tests performed, significance levels were defined at adjusted alpha 10%. Genomic inflation factors (GIF) were estimated for the two pQTL analyses using *estlambda* function available in the GenABEL R package [21]. Pairwise linkage disequilibrium (LD) was calculated with LDlink, a web-based application using 1000 Genome phase 3 data [22].

### Statistical analyses

Association between circulating ANGPTL3 and clinical variables (BMI, fasting glucose and insulin levels, total lipid levels, C-reactive protein (CRP) levels) was performed using a linear model, adjusting for center, age, gender, and baseline BMI. SNP effects were tested as additive effects. In the analyses of data from the weight loss period, models were adjusted for change in BMI. Adjustment for multiple testing was performed applying a Bonferroni correction considering tests performed on data available at baseline and during the LCD intervention separately. Statistical analyses were performed using R version 3.2.3.

## Results

### Baseline characteristics

In total, 769 participants from the DiOGenes study were included in the analyses. The baseline characteristics are described in Table 1 and have been extensively discussed in previous DiOGenes publications [10, 23, 24]. Briefly, participants were on average 41 years of age, with baseline BMI of 34.5 ± 4.9 kg/m^2^ (mean ± sd) and were non-diabetics (mean glucose levels = 5.12 ± 0.74 mmol/l and insulin levels = 11.48 ± 8.57 μIU/ml). After the weight loss period, the average BMI was decreased to 30.7 ± 4.5 kg/m^2^, and glycemic profiles improved to 4.82 ± 0.54 mmol/l for fasting glucose and 8.15 ± 6.12 μIU/ml for insulin.

**Table 1:** Participant characteristics

Variable	Baseline		Change during weight loss	
	*n*	Mean ± sd	*n*	Mean ± sd
Gender (M/F)	769	263/506	–	–
Age (years)	769	41.28 ± 6.22	–	–
BMI (kg/m^2^)	762	34.54 ± 4.88	638	3.80 ± 1.12
Total cholesterol (mmol/l)	714	4.89 ± 1.01	620	0.66 ± 0.76
HDL-C (mmol/l)	716	1.20 ± 0.33	624	0.08 ± 0.23
LDL-C (mmol/l)	711	3.07 ± 0.88	616	0.45 ± 0.64
TG (mmol/l)	705	1.35 ± 0.65	611	0.31 ± 0.58
FFA (μmol/l)	630	654.9 ± 333.2	545	− 55.3 ± 368.4
Glucose (mmol/l)	701	5.12 ± 0.74	606	0.29 ± 0.60
Insulin (μIU/ml)	683	11.48 ± 8.57	541	3.89 ± 5.60
CRP (mg/l)	698	4.30 ± 3.90	594	1.05 ± 2.55
ANGPTL3 (RFU)	567	349.2 ± 122.0	539	1.74 ± 85.8
ALT (RFU)	594	5493.6 ± 2399.9	543	6.55 ± 204.3
AST (RFU)	594	7867.6 ± 2581.6	543	− 0.93 ± 218.5
Adiponectin (RFU)	594	1430.8 ± 555.4	543	− 0.87 ± 196.7
Fetuin A (RFU)	594	1029.2 ± 190.7	543	− 4.66 ± 213.7
Fetuin B (RFU)	594	4026.6 ± 1342.0	543	2.66 ± 128.9
CK-18 (RFU)	594	222.0 ± 916.1	543	− 4.08 ± 123.2

*ALT* alanine aminotransferase, *ANGPTL3* angiopoietin-like protein 3, *AST* aspartate aminotransferase, *BMI* body mass index, *CK-18* cytokeratin 18, *CRP* C-reactive protein, *FFA* free fatty acids, *HDL-C* high-density lipoprotein cholesterol, *LDL-C* low-density lipoprotein cholesterol, *sd* standard deviation, *TG* triglycerides

### Circulating ANGPTL3 and clinical measurements

During the weight loss period, ANGPTL3 plasma concentration was marginally associated with weight loss (*p* = 0.056, see Table 2). Furthermore, ANGPTL3 concentration was positively associated with TG concentration (*p* = 0.02) and with fasting insulin levels (*p* = 0.04). For both variables, the associations were independent of weight loss. However, these associations were not significant after adjustment for multiple testing. For other variables (total cholesterol, HDL-C, LDL-C, FFA, glucose, and CRP), there were no significant associations between ANGPTL3 and their concentration at baseline or changes during the weight loss period (Table 2).

**Table 2:** Plasma ANGPTL3 and association with BMI and lipid profile

Variable	Baseline		Weight loss period	
	*β* (95%CI)	*p*	*β* (95%CI)	*p*
BMI (kg/m^2^)	0.00 (− 0.44;0.45)	0.986	6.39 (− 0.18;13.0)	0.056
Total cholesterol (mmol/l)	0.45 (− 1.75;2.69)	0.691	6.29 (− 3.11;15.7)	0.189
HDL-C (mmol/l)	− 3.62 (− 10.4;3.67)	0.322	− 21.2 (− 55.30;12.8)	0.221
LDL-C (mmol/l)	− 0.15 (− 2.60;2.37)	0.906	4.81 (− 6.17;15.8)	0.390
TG (mmol/l)	3.21 (− 0.34;6.89)	0.077	15.7 (2.15;29.2)	0.023
FFA (μmol/l)	0.01 (− 0.00;0.01)	0.134	0.00 (− 0.03;0.02)	0.728
Glucose (mmol/l)	− 0.35 (− 3.67;3.08)	0.839	− 8.13 (− 20.36;4.10)	0.193
Insulin (μIU/ml)	0.10 (− 0.18;0.37)	0.485	1.54 (0.06;3.02)	0.042
CRP (mg/l)	0.28 (− 0.35;0.91)	0.383	1.89 (− 0.89;4.66)	0.183

Coefficient (*β*), corresponding 95% confidence intervals, and associated *p* value from a linear regression are provided. Data are presented as back-transformed *β*-coefficients in percent with regard to results at baseline. Thus, an increase in ANGPTL3 of 1 RFU results in *β* (95%CI) percent change of the given variable. The regression models were adjusted for center, age, gender, and BMI. Models with data from the weight loss period were adjusted for the change in BMI due to the weight loss

*CI* Confidence interval, *CRP* C-reactive protein, *FFA* free fatty acids, *HDL-C* high-density lipoprotein cholesterol, *LDL-C* low-density lipoprotein cholesterol, *TG* triglycerides

### Circulating ANGPTL3 and liver markers

The association between ANGPTL3 and plasma levels of specific liver markers (AST, ALT, adiponectin, fetuin A and B, and CK-18) were tested (Table 3). We observed a strong positive association between circulating ANGPTL3 and CK-18 both at baseline (*p* = 1.03 × 10^−7^) and during the weight loss period (*p* = 1.47 × 10^−13^). Significant association was also seen between changes in AST and ANGPTL3 levels during weight loss intervention (*p* = 0.004). All these associations remained significant, even after adjustment for multiple testing. During weight loss, adiponectin displayed marginal association with ANGPTL3 (with nominal *p* value = 0.03; Bonferroni-adjusted *p* value = 0.18 and FDR-adjusted *p* value = 0.06).

**Table 3:** Plasma ANGPTL3 and association with liver markers

Liver markers	Function and association with liver steatosis	Baseline		Weight loss period	
		*β* (95%CI)	*p*	*β* (95%CI)	*p*
ALT (RFU)	Aminotransferase. High levels in liver. Marker of hepatocellular damage (↑).	− 0.78 (− 2.88; 1.31)	0.463	0.02 (− 0.02; 0.05)	0.405
AST (RFU)	Aminotransferase. High levels in the liver, heart, and muscle (↑).	− 1.00 (− 3.04; 1.04)	0.336	− 0.05 (− 0.08; − 0.02)	*0.004*
Adiponectin (RFU)	Induce hepatic fatty acid oxidation, inhibits fatty acid synthesis, and suppress TNF-α production in the liver (↓).	1.51 (− 0.53; 3.56)	0.150	0.04 (0.00; 0.08)	0.030
Fetuin A (RFU)	Glycoprotein produced predominantly by the liver. Inhibitor of the insulin receptor tyrosine kinase (↑).	− 0.97 (− 3.01; 1.06)	0.347	− 0.01 (− 0.04; 0.02)	0.551
Fetuin B (RFU)	Shares 22% sequence similarity with fetuin A. Linked to inflammation and insulin resistance (↑).	0.43 (− 1.70; 2.56)	0.692	0.03 (− 0.03; 0.08)	0.347
CK-18 (RFU)	Activation of caspase 3 (apoptosis) results in cleavage of CK-18, the major intermediate filament in hepatocytes (↑).	5.90 (3.82; 7.99)	*1.03 × 10* ^*−7*^	0.21 (0.15; 0.26)	*1.47 × 10* ^*−13*^

Coefficient (*β*), corresponding 95% confidence intervals, and associated *p* value from a linear regression are provided (in italics, *p* values passing Bonferroni correction). Data are presented as back-transformed *β*-coefficients in percent with regard to results at baseline. Thus, an increase in ANGPTL3 of 1 RFU results in *β* (95%CI) percent change of the given variable. In italics, *p* values passing Bonferroni correction (*p* < 0.05/6 = 0.0083). The regression models were adjusted for center, age, gender, and BMI. Models with data from the weight loss period were adjusted for the change in BMI due to the weight loss

*ALT* alanine aminotransferase, *AST* aspartate aminotransferase, *CI* Confidence interval, *CK-18* cytokeratin 18, *RFU* relative fluorescence units, *TNF-α* tumor necrosis factor α

### ANGPTL3 pQTL analyses

**Fig. 1:**
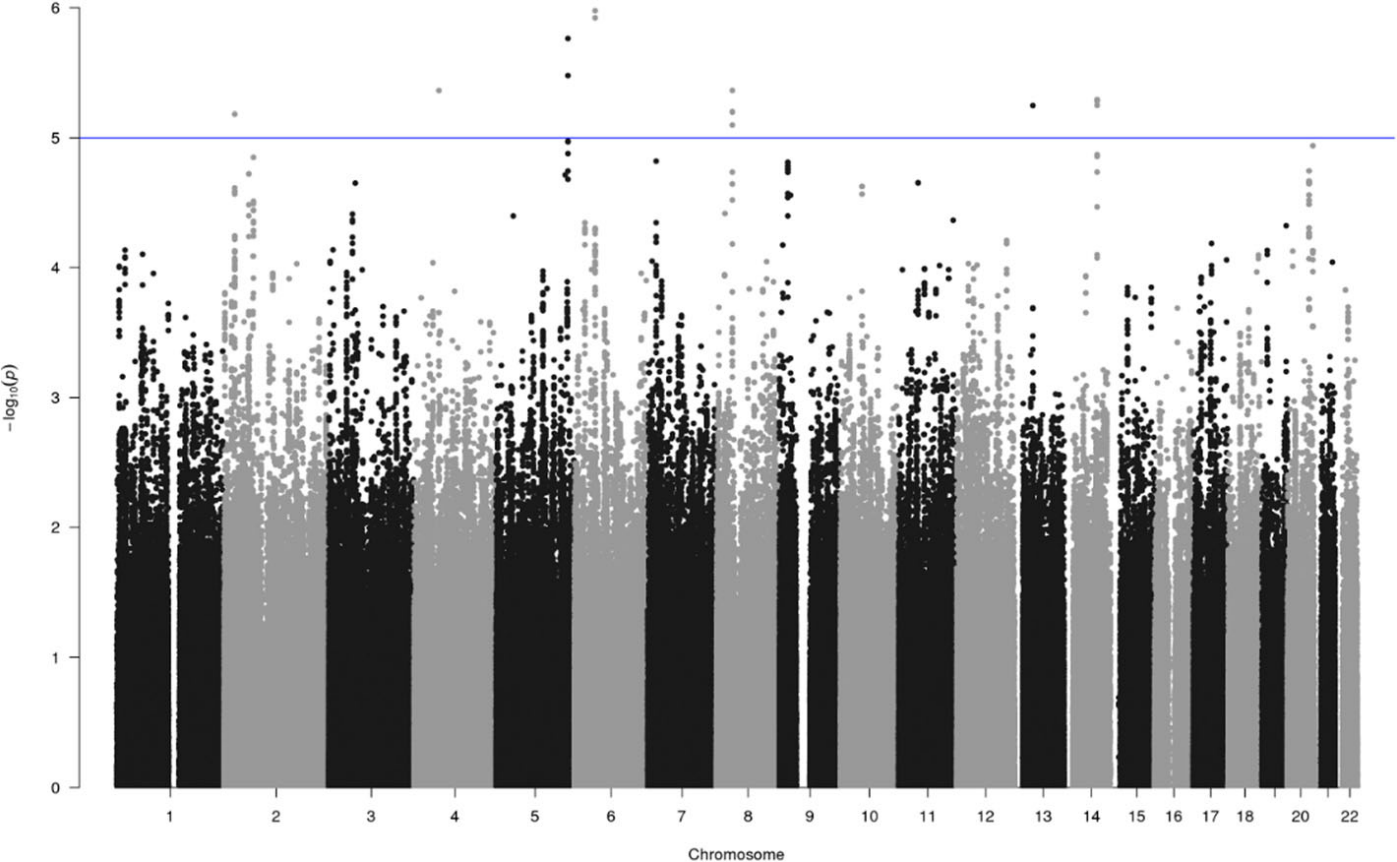
pQTL analysis of SNPs associated with circulating ANGPTL3 at baseline. Manhattan plot of pQTL analysis of SNPs associated with circulating ANGPTL3 at baseline. Each SNP is indicated by a black or a gray dot. They are arranged by chromosomal location (*x*-axis). The *y*-axis illustrates the level of statistical significance measured by the negative log of the corresponding *p* value for each SNP. The blue line represents suggestive association (*p* < 1 × 10^−5^)

**Fig. 2:**
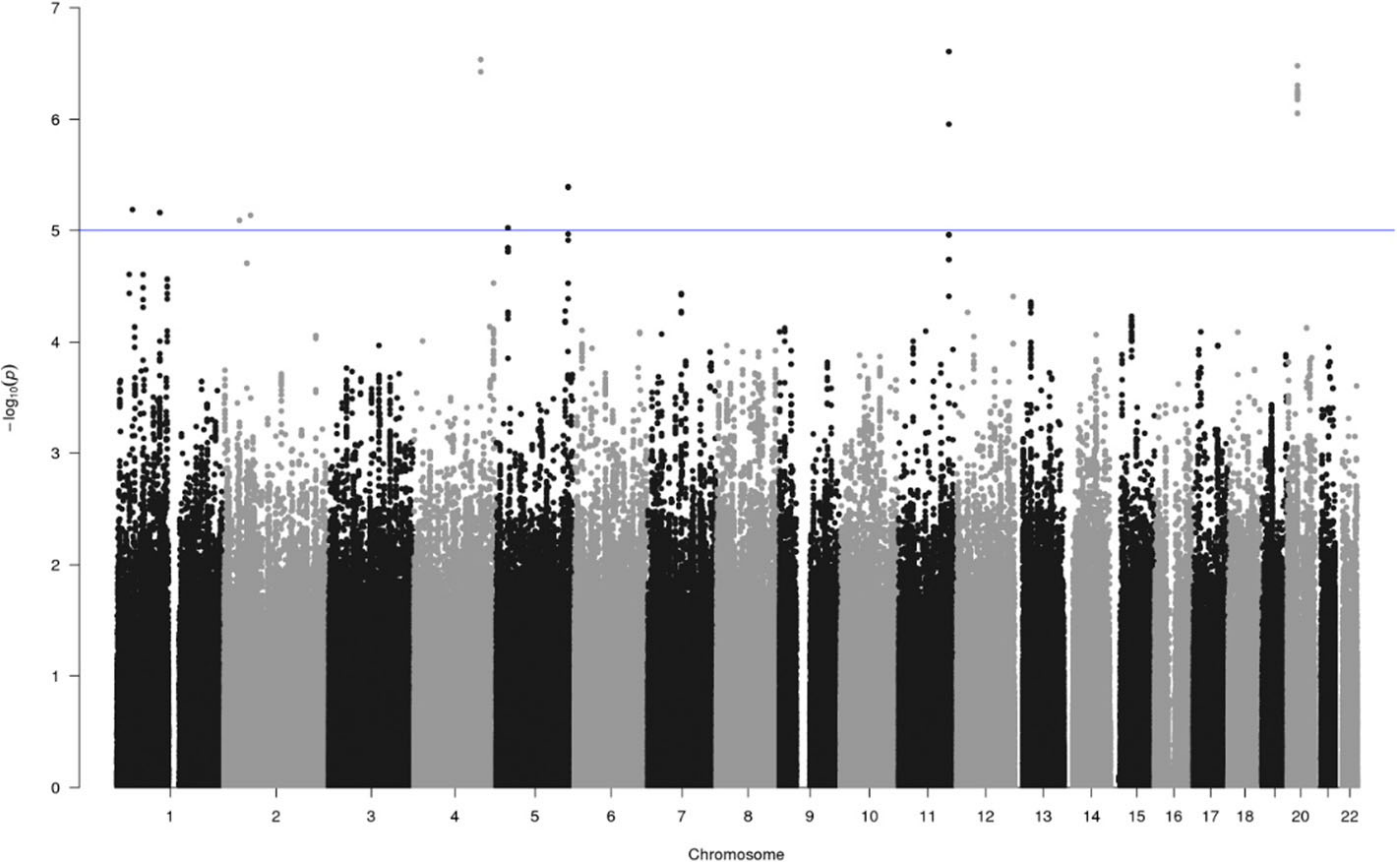
pQTL analysis of SNPs associated with the change in circulating ANGPTL3 during weight loss. Manhattan plot of pQTL analysis of SNPs associated with the change in circulating ANGPTL3 during weight loss. Each SNP is indicated by a black or a gray dot. They are arranged by chromosomal location (*x*-axis). The *y*-axis illustrates the level of statistical significance measured by the negative log of the corresponding *p* value for each SNP. The blue line represents suggestive association (*p* < 1 × 10^−5^)

Furthermore, we investigated the possible link between circulating ANGPTL3 levels (at baseline and changes during LCD) and genetic markers. We thus performed genome-wide pQTL analyses testing more than 4 million common variants (see the “Methods” section). The results are shown as Manhattan plots in Figs. 1 and 2, respectively for the baseline and LCD pQTLs. Baseline pQTL analysis did not highlight any genome-wide significant signals (at adjusted alpha < 0.10).

**Fig. 3:**
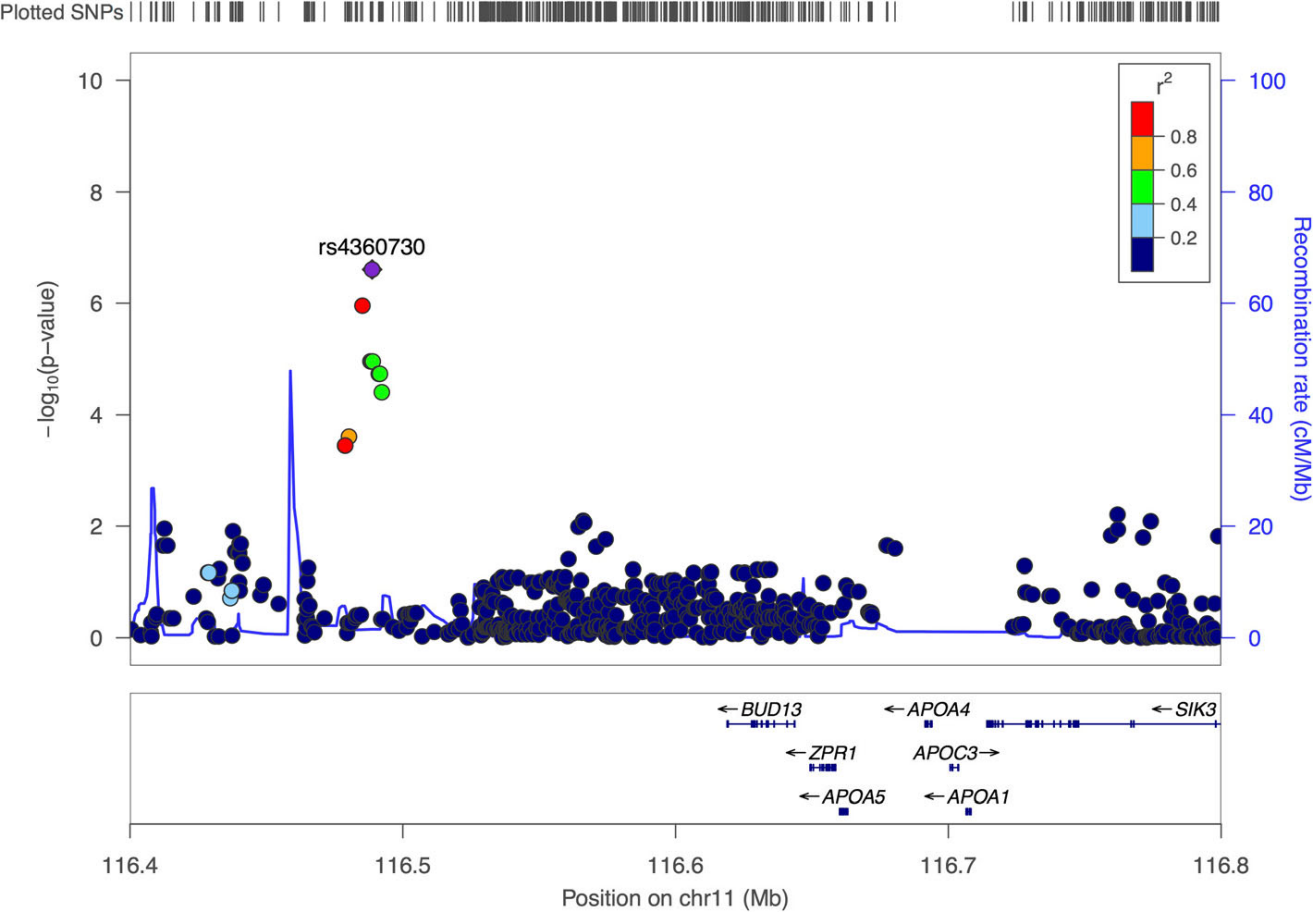
pQTL association signals at baseline in the region of surrounding rs4360730 and rs74234276 and *BUD12*, *APOA4*, *APOA5*, and *ZNF259* (*ZPR1*) genes cluster. Association plot produced using LocusZoom software for SNPs associated to ANGPTL3 protein level at baseline. SNPs’ *p* values are plotted after −log10 transformation with scale on the *y*-axis and colors reflect pairwise linkage disequilibrium with the most associated SNP in the region (purple dot) based on the 1000 genomes EUR data set

**Fig. 4:**
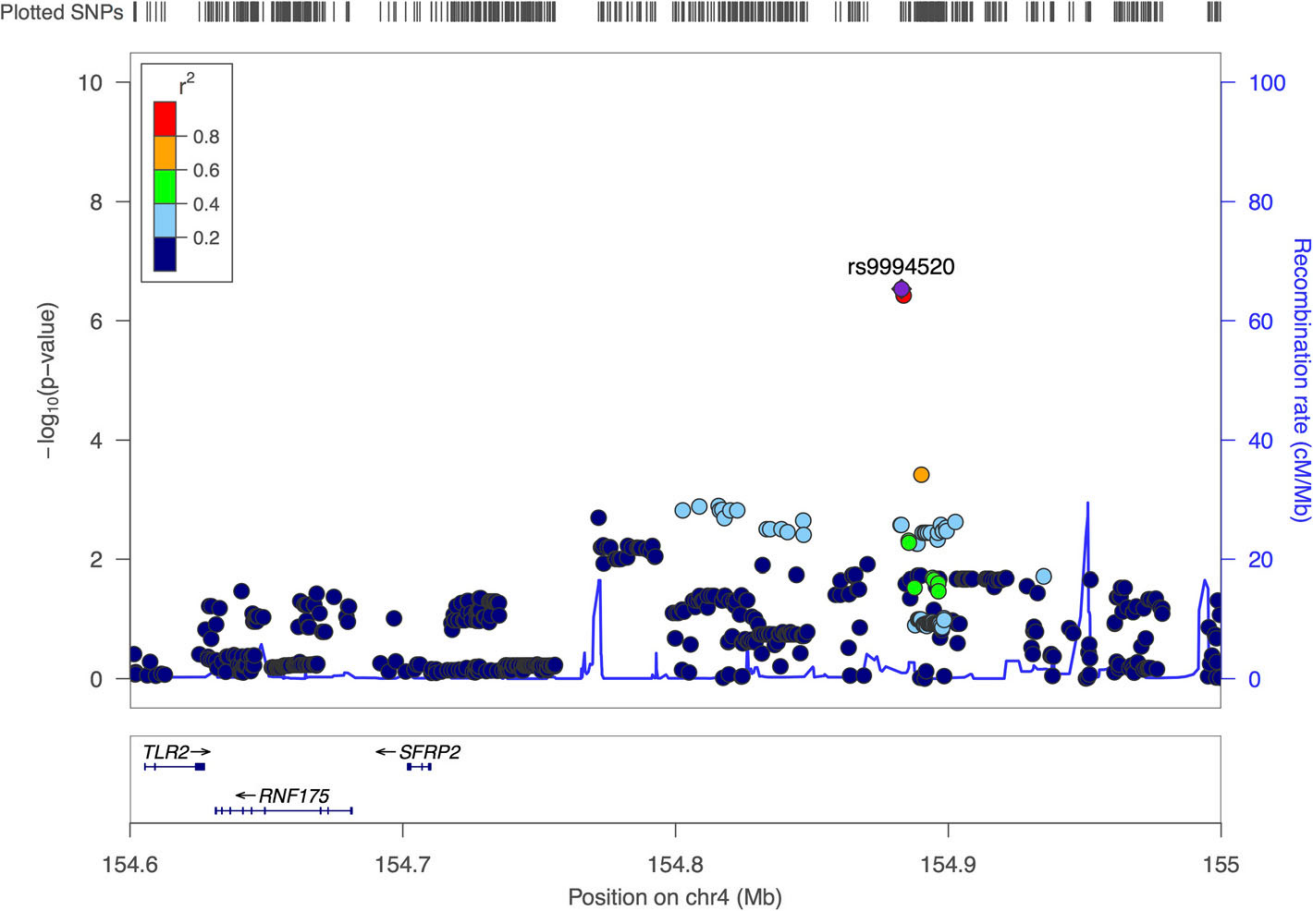
pQTL association signals during weight loss in the region surrounding rs9994520 and SFRP2 gene. Association plot produced using LocusZoom software for SNPs associated to ANGPTL3 protein level change during weight loss intervention. SNPs’ *p* values are plotted after −log10 transformation with scale on the *y*-axis and colors reflect pairwise linkage disequilibrium with the most associated SNP in the region (purple dot) based on the 1000 genomes EUR data set

The top SNPs (with nominal *p* < 1 × 10^− 4^) are presented in Table 4. However, in the LCD pQTL, three variants were considered genome-wide significant (Table 5). The two first SNPs, rs4360730 (NC_000011.9:g.116488748T>C) and rs74234276 (NC_000011.9:g.116488753G>A) are in perfect LD (*R*^2^ = 1) and localized within an intergenic region located 120 kb downstream from *BUD13* gene (Fig. 3). This gene belongs to a gene cluster together with *APOA4*, *APOA5*, and *ZNF259*. The third SNP, rs9994520 (NC_000004.11:g.154882844G>C), is located 170 kb upstream from *SFRP2* gene (Fig. 4). For both pQTL analyses, no significant *p* value inflation was observed (GIF were 1.00 and 0.99, respectively for baseline and weight loss pQTL, Additional file 1: Figure S1 and Additional file 2: Figure S2). This indicated no bias due to population substructure.

**Table 4:** SNPs associated with circulating ANGPTL3 at baseline (*p* < 1 × 10^−5^)

SNP	Chr	Position (bp)	A1	A2	MAF	Coef.	se	*p* value
rs36000763	6	48,687,252	A	G	0.064	0.202	0.041	1.06 × 10^−6^
rs41528149	6	48,728,020	T	C	0.065	0.200	0.041	1.20 × 10^−6^
rs13185453	5	166,370,819	A	G	0.143	0.143	0.030	1.73 × 10^−6^
rs35976153	5	166,365,612	T	C	0.146	0.136	0.029	3.34 × 10^−6^
rs12334611	8	37,045,986	C	T	0.072	0.182	0.040	4.35 × 10^−6^
rs1277307	4	57,896,699	G	T	0.097	0.162	0.035	4.36 × 10^−6^
rs76263326	14	76,627,919	C	T	0.100	0.151	0.033	5.12 × 10^−6^
rs12100883	14	76,628,814	T	C	0.100	0.151	0.033	5.12 × 10^−6^
rs1900121	14	76,629,481	T	C	0.100	0.151	0.033	5.12 × 10^−6^
rs3783998	14	76,632,781	G	T	0.101	0.151	0.033	5.26 × 10^−6^
rs4903381	14	76,635,675	C	G	0.100	0.151	0.033	5.67 × 10^−6^
rs17521181	13	42,828,989	A	T	0.089	0.164	0.036	5.69 × 10^−6^
rs4739476	8	37,046,991	A	G	0.070	0.184	0.041	6.33 × 10^−6^
rs74528305	2	25,066,379	G	T	0.077	0.177	0.039	6.57 × 10^−6^
rs74571086	8	37,049,958	G	A	0.070	0.184	0.041	7.97 × 10^−6^

Results from the association between SNPs and ANGPTL3 level at baseline

*A1 and A2* the minor and major alleles, *bp* basepair, *Chr* chromosome, *Coef* estimated association coefficient, *MAF* minor allele frequency, *se* standard error, *SNP* single nucleotide polymorphism

**Table 5:** SNPs associated with change in circulating ANGPTL3 during weight loss intervention (*p* < 1 × 10^−5^)

SNP	Chr	Position (bp)	A1	A2	MAF	Coef.	se	*p* value
rs4360730*	11	116,488,748	C	T	0.057	0.166	0.032	2.48 × 10^−7^
rs74234276*	11	116,488,753	A	G	0.057	0.166	0.032	2.48 × 10^−7^
rs9994520*	4	154,882,844	G	C	0.304	0.084	0.016	2.93 × 10^−7^
rs113794502	20	23,631,539	G	C	0.237	− 0.091	0.018	3.33 × 10^−7^
rs7661078	4	154,883,600	A	G	0.302	0.083	0.016	3.78 × 10^−7^
rs55656752	20	23,631,510	A	T	0.236	− 0.090	0.018	5.01 × 10^−7^
rs112213361	20	23,631,523	A	G	0.236	− 0.090	0.018	5.01 × 10^−7^
rs73102376	20	23,633,232	T	C	0.234	− 0.090	0.018	5.53 × 10^−7^
rs73102379	20	23,633,245	C	T	0.234	− 0.090	0.018	5.53 × 10^−7^
rs73102363	20	23,631,599	G	C	0.236	− 0.089	0.018	5.78 × 10^−7^
rs73102364	20	23,631,602	G	A	0.236	− 0.089	0.018	5.78 × 10^−7^
rs73102366	20	23,631,654	T	C	0.236	− 0.089	0.018	5.78 × 10^−7^
rs60143382	20	23,631,067	A	G	0.236	− 0.090	0.018	5.84 × 10^−7^
rs55724037	20	23,631,068	C	T	0.236	− 0.090	0.018	5.84 × 10^−7^
rs58578197	20	23,631,309	C	T	0.236	− 0.090	0.018	5.84 × 10^−7^
rs112950650	20	23,632,409	G	A	0.235	− 0.089	0.018	6.05 × 10^−7^
rs8116240	20	23,632,730	T	C	0.235	− 0.089	0.018	6.05 × 10^−7^
rs8122969	20	23,632,847	C	T	0.235	− 0.089	0.018	6.05 × 10^−7^
rs8124308	20	23,633,094	C	T	0.235	− 0.089	0.018	6.17 × 10^−7^
rs8122922	20	23,632,776	C	T	0.236	− 0.089	0.018	6.50 × 10^−7^

Results from the association between SNPs and ANGPTL3 protein level change during weight loss intervention

*A1 and A2* the minor and major alleles, *bp* basepair, *Chr* chromosome, *Coef* estimated association coefficient, *MAF* minor allele frequency, *se* standard error, *SNP* Single nucleotide polymorphism

*SNPs with adjusted *p* value < 0.10 upon the SLIDE (permutation) *p* value adjustments

### Association between genetic markers and liver markers

Based on the pQTL results, rs4360730 and rs9994520 were chosen for further analysis. Specifically, we assessed whether the two liver markers (CK-18 and AST) associated with ANGPTL3 levels were also under genetic control. rs74234276 was not included due to complete LD with rs4360730. Regarding the rs4360730 SNP, we observed a significant association with CK-18 during weight loss period (with nominal *p* = 0.007 and Bonferroni adjusted *p* = 0.028, see Additional file 3: Table S1) and marginal association at baseline (*p* = 0.086). Effect size per genotype groups are indicated in Additional file 3: Table S1. Association tests with ALT levels did not reveal any significant effect of rs4360730. rs4360730 was not previously identified in published GWAs (EBI GWAs catalog, 01/01/2018 release) nor was it previously identified as an eQTL SNP in GTEX (release 7) [25, 26]. For rs9994520, we did not observe any significant association with CK-18 or ALT levels (at baseline and changes during LCD, see Additional file 3: Table S2).

## Discussion

In the current study, we addressed the link between circulating ANGPTL3 levels and clinical improvements (weight, plasma lipid, and insulin profile) during LCD in a large clinical study. We assessed the link between ANGPTL3 and liver markers (released in circulation), and whether ANGPTL3 levels were under genetic control. Finally, we investigated the contribution from genetic markers modulating ANGPTL3 levels on liver markers themselves.

We observed a positive association between circulating ANGPTL3 and TG concentration following weight loss. However, this association was modest and did not remain, when correcting for multiple testing. In general, results on the relationship between circulating ANGPTL3 concentration and plasma lipids in humans are inconsistent [27–32]. In contrast to what could be expected, Robciuc and colleagues reported a negative correlation between ANGPTL3 and TG concentration [31]. This correlation did not remain significant after adjusting for HDL-C and apolipoprotein concentrations. A large study including 1770 participants of European Caucasian ancestry did not observe a correlation between plasma ANGPTL3 and concentration of TG [32]. However, they did report positive correlations between ANGPTL3 concentrations and LDL-C, HDL-C, and total cholesterol. Despite conflicting results concerning the relationship between ANGPTL3 and lipid parameters in humans, there is a consensus about the physiological role of ANGPTL3 regarding inhibition of LPL. But the functional evidence is derived from animal studies [33, 34] and the exact inhibitory mechanisms of ANGPTL3 on LPL in humans are not fully understood. Earlier findings indicate that cleavage is crucial for the function of ANGPTL3. The N-terminal fragment containing the coiled coil domains of the protein is more efficient in inhibiting LPL than the full-length ANGPTL3 [2]. In this study, we used a detection method based on protein binding of aptamers, which are reported to have many advantages, compared to antibodies [35]. However, in this and several other studies, the methods used for detecting ANGPTL3 cannot distinguish between the different fragments of the protein, nor post-translational modification. It is suggested that the functional fraction of ANGPTL3 might not be found in circulation, but exists bound to the endothelial surface of the adipose tissue, cardiac muscle, and skeletal muscle for LPL-mediated lipolysis [36]. This further specifies the need of an improved understanding regarding the LPL inhibitory function of ANGPTL3 and further improvement of the methods to detect and quantify the fragments of the protein.

A study reported that the ANGPTL8 is the rate-limiting protein for the activity of ANGPTL3 [37]. Co-expression of ANGPTL3 and ANGPTL8 in cultured hepatocytes resulted in the appearance of a 33-kDa-sized protein corresponding to the N-terminal domain of ANGPTL3, whereas only full-length ANGPTL3 were detected in cells that did not express ANGTPL8. ANGPTL8 was not assayed on the Somalogic panel, and it was not possible to study the relationship with ANGPTL3 within the DiOGenes study. However, recent in vivo studies have further indicated that ANGPTL3 and ANGPTL8 cooperate in the regulation of plasma TG levels [38, 39]. Davies and colleagues demonstrated that ANGPTL3 and ANGPTL8 as a complex exhibited a greatly enhanced ability to bind LPL compared to either protein alone. This complex was formed more efficiently, when the two proteins were co-expressed [39]. This has led to the suggestion of interplay between ANGPTL3, ANGPTL4, and ANGPTL8 in the regulation of lipid metabolism [40, 41]. ANGPTL8 is induced by feeding and possibly activates the inhibitory effects of ANGPTL3 on LPL in cardiac and skeletal muscles, directing circulating TG to the adipose tissue for storage. In this study, the concentration of circulating ANGPTL3 and lipid parameters were measured in a fasted state, which could explain the lack of significant associations. It is likely that an ANGPTL3 response is only observed post-prandial, and thus, a meal-test challenge would be required to study the dynamics of ANGPTL3. ANGPTL4 is very similar to ANGPTL3 both in structure and in function and is induced by fasting and might inhibit LPL in adipose tissue during energy restriction, directing TG to cardiac and skeletal muscle for oxidation [40, 41].

Consistent with the conflicting results regarding ANGPTL3 and lipid metabolism, the link between ANGPTL3 and glucose metabolism remains unclear [42, 43]. Our results showed a marginal association between circulating ANGPTL3 and fasting insulin concentrations. The mechanisms by which ANGPTL3 influence the insulin remains unclear, but there might be a potential role of the protein to indirectly regulate glucose metabolism.

We found a strong positive association between changes in ANGPTL3 levels and CK-18, together with a negative association between changes in ANGPTL3 and AST, both independently of weight loss. CK-18 is the major intermediate filament protein in the liver. Circulating CK-18 is associated with apoptotic cell death of hepatocytes, and several studies have demonstrated the elevation of CK-18 in the context of NASH and hepatic inflammation [44]. AST is a transaminase enzyme dependent on pyridoxal phosphate and important in the amino acid metabolism. It is present as both cytoplasmic and mitochondrial isoforms. In this study, we measured the cytoplasmic isoform, which independently is a marker of tissue injury. High levels of circulating AST is not exclusively related to the liver steatosis, but could also indicate diseases affecting other organs, as AST is found in high concentrations in the liver, heart, skeletal muscle, and kidney [45, 46]. To our knowledge, only one human study has analyzed circulating ANGPTL3 concentration in relation to liver steatosis. This study found that ANGPTL3 concentration was significantly and independently associated with NASH, but not in patients with simple steatosis [9]. Szalowska et al. induced inflammation in human liver tissues in vitro and identified ANGPTL3 as a biomarker associated with liver diseases [47]. Together with our results regarding CK-18, it could indicate that an increase in plasma ANGPTL3 concentration is the result of liver inflammation or that ANGPTL3 plays a role in the development of the diseased condition. Due to the controversy of non-invasive biomarkers as measurement of liver diseases, additional studies should include actual liver biopsies to further evaluate the role of ANGPTL3 in liver steatosis.

Our pQTL study highlighted SNPs that were modulating changes in circulating ANGPTL3 during the weight loss period, of which one locus also seemed to modulate CK-18 levels. Specifically, these pQTL studies revealed three common genetic variants (rs4360730, rs74234276, and rs9994520) associated with circulating ANGPTL3. SNPs rs4360730 and rs74234276 are located near the *APOA4-APOA5-ZNF259-BUD13* gene cluster locus at the chromosome region 11q23.3; and are in perfect LD. Several genetic variants in this region have already been associated to hyperlipidemia [48], serum lipid levels [49], risk of developing metabolic syndrome [50], and plasma TG level [51]. *APOA4* and *APOA5* encode apolipoproteins involved in lipid metabolism [52]. *ZNF259* encodes zinc finger protein, a regulatory protein that is involved in cell proliferation and signal transduction. *BUD13* encodes for BUD13 homolog protein, which is a subunit in the retention and splicing (RES) complex that affects nuclear pre-mRNA retention. However, the exact function of ZNF259 and BUD13 in lipid mechanisms is unclear [48]. The region is an interesting target knowing that ANGPTL3 regulates plasma lipid levels and is a potential therapeutic target to treat combined hyperlipidemia [53]. The SNPs in this region, rs4360730 and rs74234276, are *trans*-acting genetic variants, probably working as distant regulators of ANGPTL3 through mechanisms of the *APOA4-APOA5-ZNF259-BUD13* gene cluster. We further demonstrated that CK-18 levels at baseline and during the weight loss period were under genetic control by the rs4360730 SNP.

The rs9994520 SNP is located near the *SFRP2* gene. This gene encodes the secreted Frizzled-related protein 2, which operates as soluble modulators of Wnt signaling. The functional relationship between ANGPTL3 and SFRP2 is not known. However, *SFRP2* has been associated to adipose tissue mass and may play a role in adipose angiogenesis of which angiopoietin-like proteins are regulation key factors [54–56].

Interestingly, the identified pQTLs affecting circulating ANGPTL3 during the weight loss intervention were not detectable at baseline. This is consistent with our recent large-scale pQTL study on 1129 proteins [17], where the identified pQTL during LCD could not be identified at baseline. This can be explained by effect size consideration (statistical power): very large sample size would be required to identify potential baseline pQTL. By contrast, a clinical intervention (such as LCD) would induce drastic metabolic and physiological changes, thus would lead to very large effect sizes and thereby significantly improve our ability to detect pQTLs associated with such drastic shift in homeostasis [17].

## Conclusions

In conclusion, we uncover genetic regulators of circulating ANGPTL3 during LCD and the link with markers of liver function. We report several *trans-*acting pQTL on changes in circulating ANGPTL3 during LCD. These pQTLs were not detectable at baseline, suggesting a change in the regulation of ANGPTL3 due to calorie restriction. It was not possible to clarify the controversy regarding the function of ANGPTL3 in lipid metabolism as we found a very marginal association with total lipid levels. However, our data suggest strong associations with specific liver markers (CK-18 and AST). These observations are supported by the identification of pQTL signals that affect ANGPTL3 levels during the weight loss period. Our analysis also suggests an extended function of ANGPTL3 in the development of liver steatosis and shows a common genetic regulation for both ANGPTL3 and markers of liver function.

## Additional files

Additional file 1: **Figure S1.** QQ plot of the relationship between expected and observed distribution at baseline. Quantile-quantile plot of baseline data. The relationship between observed (y-axis) and expected (x-axis) distribution. The statistical significance is measured by the negative log of the corresponding *p*-value for each SNP. (JPEG 92 kb)

Additional file 2: **Figure S2.** QQ plot of the relationship between expected and observed distribution during weight loss period. Quantile-quantile plot for the analysis of the weight loss period. The relationship between observed (y-axis) and expected (x-axis) distribution. The statistical significance is measured by the negative log of the corresponding *p*-value for each SNP. (JPEG 94 kb)

Additional file 3: **Table S1.** Effect of rs4360730 on BMI, Lipid Profile and Liver Markers. Table S2 Effect of rs9994520 on BMI, Lipid Profile and Liver Markers. (DOCX 21 kb)

## Abbreviations

ALT: Alanine aminotransferase
ANGPTL3/4/8: Angiopoietin-like protein 3/4/8
ANGPTLs: Angiopoietin-like proteins
AST: Aspartate aminotransferase
BMI: Body mass index
CK-18: Cytokeratin 18
CRP: C-reactive protein
FDR: False discovery rate
FFA: Free fatty acids
GI: Glycemic index
GIF: Genomic inflation factor
HDL-C: High-density lipoprotein cholesterol
LCD: Low-calorie diet
LD: Linkage disequilibrium
LDL-C: Low-density lipoprotein cholesterol
LPL: Lipoprotein lipase
NASH: Non-alcoholic steatohepatitis
pQTL: Protein quantitative trait locus
RES complex: Retention and splicing complex
RFU: Relative fluorescence units
sd: Standard deviation
se: Standard error
SLIDE: Sliding-window method for Locally Inter-correlated markers with asymptotic Distribution Errors corrected
SNPs: Single nucleotide polymorphisms
TG: Triglycerides

## References

[1] Grundy SM (2008). Metabolic syndrome pandemic. Arterioscler Thromb Vasc Biol.

[2] Ono M (2003). Protein region important for regulation of lipid metabolism in angiopoietin-like 3 (ANGPTL3): ANGPTL3 is cleaved and activated in vivo. J Biol Chem.

[3] Conklin D (1999). Identification of a mammalian angiopoietin-related protein expressed specifically in liver. Genomics.

[4] Shan L (2009). The angiopoietin-like proteins ANGPTL3 and ANGPTL4 inhibit lipoprotein lipase activity through distinct mechanisms. J Biol Chem.

[5] Koishi R (2002). Angptl3 regulates lipid metabolism in mice. Nat Genet.

[6] Romeo S (2009). Rare loss-of-function mutations in ANGPTL family members contribute to plasma triglyceride levels in humans. J Clin Invest.

[7] Musunuru K (2010). Exome sequencing, ANGPTL3 mutations, and familial combined hypolipidemia. N Engl J Med.

[8] Minicocci I (2012). Mutations in the ANGPTL3 gene and familial combined hypolipidemia: a clinical and biochemical characterization. J Clin Endocrinol Metab.

[9] Yilmaz Y (2009). Serum concentrations of human angiopoietin-like protein 3 in patients with nonalcoholic fatty liver disease: association with insulin resistance. Eur J Gastroenterol Hepatol.

[10] Larsen TM (2010). Diets with high or low protein content and glycemic index for weight-loss maintenance. N Engl J Med.

[11] Larsen TM (2010). The Diet, Obesity and Genes (Diogenes) Dietary Study in eight European countries—a comprehensive design for long-term intervention. Obes Rev.

[12] Moore CS (2010). Dietary strategy to manipulate ad libitum macronutrient intake, and glycaemic index, across eight European countries in the Diogenes Study. Obes Rev.

[13] Friedewald WT, Levy RI, Fredrickson DS (1972). Estimation of the concentration of low-density lipoprotein cholesterol in plasma, without use of the preparative ultracentrifuge. Clin Chem.

[14] Gold L, et al. Aptamer-based multiplexed proteomic technology for biomarker discovery. PLoS One. 2010;5(12):e15004. 10.1371/journal.pone.0015004 PMC3000457 21165148

[15] Rohloff JC (2014). Nucleic acid ligands with protein-like side chains: modified aptamers and their use as diagnostic and therapeutic agents. Mol Ther Nucleic Acids.

[16] SOMAscan Technical White Paper. 2017 [cited 2018 02-02-2018]; Available from: http://www.somalogic.com/somalogic/media/Assets/PDFs/SSM-002-Rev-2-SOMAscan-Technical-White-Paper-3-7-15.pdf.

[17] Carayol J (2017). Protein quantitative trait locus study in obesity during weight-loss identifies a leptin regulator. Nat Commun.

[18] Das S (2016). Next-generation genotype imputation service and methods. Nat Genet.

[19] Yang J (2011). GCTA: a tool for genome-wide complex trait analysis. Am J Hum Genet.

[20] Han B, Kang HM, Eskin E (2009). Rapid and accurate multiple testing correction and power estimation for millions of correlated markers. PLoS Genet.

[21] Aulchenko YS (2007). GenABEL: an R library for genome-wide association analysis. Bioinformatics.

[22] Machiela MJ, Chanock SJ (2015). LDlink: a web-based application for exploring population-specific haplotype structure and linking correlated alleles of possible functional variants. Bioinformatics.

[23] Valsesia A (2016). Distinct lipid profiles predict improved glycemic control in obese, nondiabetic patients after a low-caloric diet intervention: the Diet, Obesity and Genes randomized trial. Am J Clin Nutr.

[24] Armenise C (2017). Transcriptome profiling from adipose tissue during a low-calorie diet reveals predictors of weight and glycemic outcomes in obese, nondiabetic subjects. Am J Clin Nutr.

[25] MacArthur J (2017). The new NHGRI-EBI Catalog of published genome-wide association studies (GWAS Catalog). Nucleic Acids Res.

[26] Consortium GT (2017). Genetic effects on gene expression across human tissues. Nature.

[27] Hatsuda S (2007). Association between plasma angiopoietin-like protein 3 and arterial wall thickness in healthy subjects. J Vasc Res.

[28] Shimamura M (2007). Angiopoietin-like protein3 regulates plasma HDL cholesterol through suppression of endothelial lipase. Arterioscler Thromb Vasc Biol.

[29] Stejskal D (2007). Angiopoietin-like protein 3: development, analytical characterization, and clinical testing of a new ELISA. Gen Physiol Biophys.

[30] Shoji T (2009). Plasma angiopoietin-like protein 3 (ANGPTL3) concentration is associated with uremic dyslipidemia. Atherosclerosis.

[31] Robciuc MR (2010). Quantitation of serum angiopoietin-like proteins 3 and 4 in a Finnish population sample. J Lipid Res.

[32] Mehta N (2014). Differential association of plasma angiopoietin-like proteins 3 and 4 with lipid and metabolic traits. Arterioscler Thromb Vasc Biol.

[33] Shimizugawa T (2002). ANGPTL3 decreases very low density lipoprotein triglyceride clearance by inhibition of lipoprotein lipase. J Biol Chem.

[34] Koster A (2005). Transgenic angiopoietin-like (angptl)4 overexpression and targeted disruption of angptl4 and angptl3: regulation of triglyceride metabolism. Endocrinology.

[35] Han K, Liang ZQ, Zhou ND (2010). Design strategies for aptamer-based biosensors. Sensors.

[36] Sonnenburg WK (2009). GPIHBP1 stabilizes lipoprotein lipase and prevents its inhibition by angiopoietin-like 3 and angiopoietin-like 4. J Lipid Res.

[37] Quagliarini F (2012). Atypical angiopoietin-like protein that regulates ANGPTL3. Proc Natl Acad Sci U S A.

[38] Haller JF (2017). ANGPTL8 requires ANGPTL3 to inhibit lipoprotein lipase and plasma triglyceride clearance. J Lipid Res.

[39] Davies BSJ, et al. ANGPTL8 promotes the ability of ANGPTL3 to inhibit lipoprotein lipase. FASEB J. 2017;31:1137–1149. 10.1016/j.molmet.2017.06.014 PMC5641604 29031715

[40] Zhang R. The ANGPTL3-4-8 model, a molecular mechanism for triglyceride trafficking. Open Biol. 2016;6(4):150272. 10.1098/rsob.150272 PMC4852456 27053679

[41] Dijk W, Kersten S (2016). Regulation of lipid metabolism by angiopoietin-like proteins. Curr Opin Lipidol.

[42] Robciuc MR (2013). Angptl3 deficiency is associated with increased insulin sensitivity, lipoprotein lipase activity, and decreased serum free fatty acids. Arterioscler Thromb Vasc Biol.

[43] Haridas PAN (2015). Regulation of angiopoietin-like proteins (ANGPTLs) 3 and 8 by insulin. J Clin Endocrinol Metab.

[44] Diab DL (2008). Cytokeratin 18 fragment levels as a noninvasive biomarker for nonalcoholic steatohepatitis in bariatric surgery patients. Clin Gastroenterol Hepatol.

[45] Giannini EG, Testa R, Savarino V (2005). Liver enzyme alteration: a guide for clinicians. CMAJ.

[46] Gowda S (2009). A review on laboratory liver function tests. Pan Afr Med J.

[47] Szalowska E (2011). Comparative analysis of the human hepatic and adipose tissue transcriptomes during LPS-induced inflammation leads to the identification of differential biological pathways and candidate biomarkers. BMC Med Genet.

[48] Aung LHH (2014). Association of the variants in the BUD13-ZNF259 genes and the risk of hyperlipidaemia. J Cell Mol Med.

[49] LHH A, et al. Association between the MLX interacting protein-like, BUD13 homolog and zinc finger protein 259 gene polymorphisms and serum lipid levels. Sci Rep. 2014;4:5565. 10.1038/srep05565 PMC5381541 24989072

[50] Lin E (2016). Association and interaction of APOA5, BUD13, CETP, LIPA and health-related behavior with metabolic syndrome in a Taiwanese population. Sci Rep.

[51] Fu Q (2015). Effects of polymorphisms in APOA4-APOA5-ZNF259-BUD13 gene cluster on plasma levels of triglycerides and risk of coronary heart disease in a Chinese Han population. PLoS One.

[52] Delgado-Lista J (2010). Effects of variations in the APOA1/C3/A4/A5 gene cluster on different parameters of postprandial lipid metabolism in healthy young men. J Lipid Res.

[53] Tikka A, Jauhiainen M (2016). The role of ANGPTL3 in controlling lipoprotein metabolism. Endocrine.

[54] Crowley RK, et al. SFRP2 is associated with increased adiposity and VEGF expression. PLoS One. 2016;11(9):e0163777. 10.1371/journal.pone.0163777 PMC5042473 27685706

[55] Courtwright A (2009). Secreted frizzle-related protein 2 stimulates angiogenesis via a Calcineurin/NFAT signaling pathway. Cancer Res.

[56] Hato T, Tabata M, Oike Y (2008). The role of angiopoietin-like proteins in angiogenesis and metabolism. Trends Cardiovasc Med.

