# Supplementary figures and images for: Analysis of circulating angiopoietin-like protein 3 and genetic variants in lipid metabolism and liver health: the DiOGenes study
Source: Genes Nutr. 2018 Apr 2;13:7. doi: 10.1186/s12263-018-0597-3 (PMC5879874; doi:10.1186/s12263-018-0597-3)

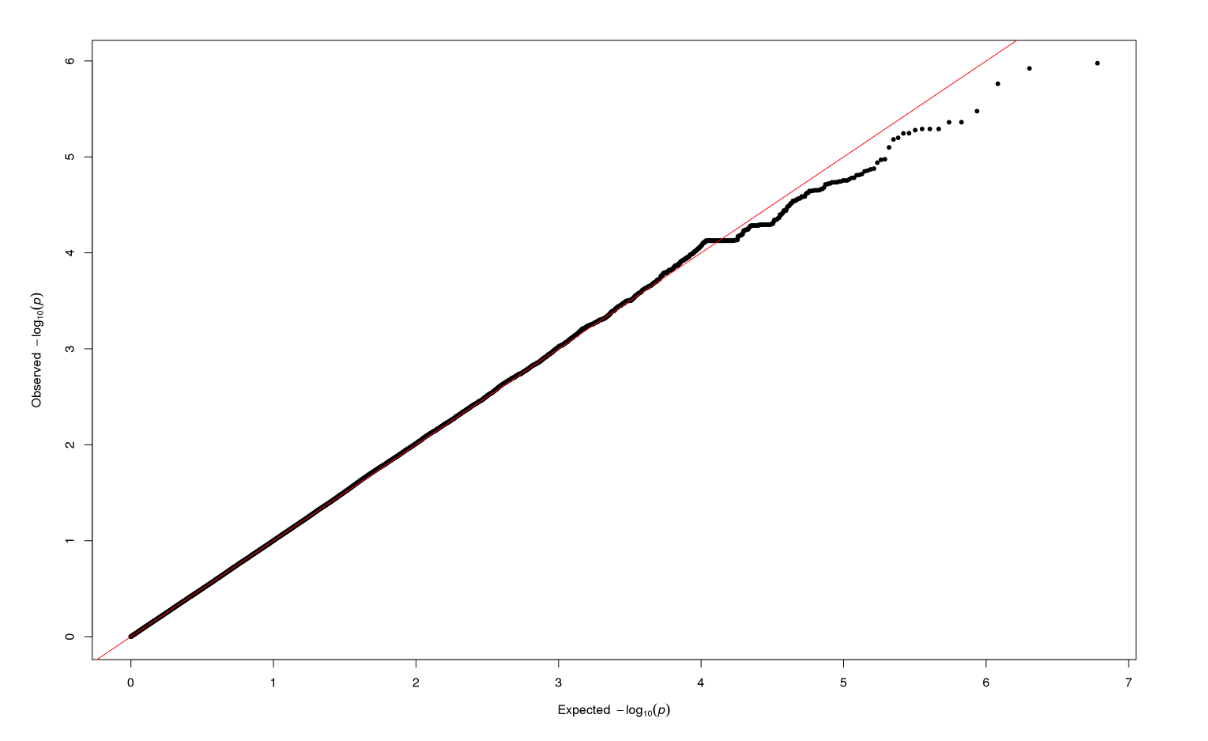

Supplement: Supplementary file 1 — Figure S1. QQ plot of the relationship between expected and observed distribution at baseline. Quantile-quantile plot of baseline data. The relationship between observed (y-axis) and expected (x-axis) distribution. The statistical significance is measured by the negative log of the corresponding p-value for each SNP. (JPEG 92 kb) [file 12263_2018_597_MOESM1_ESM.jpg]

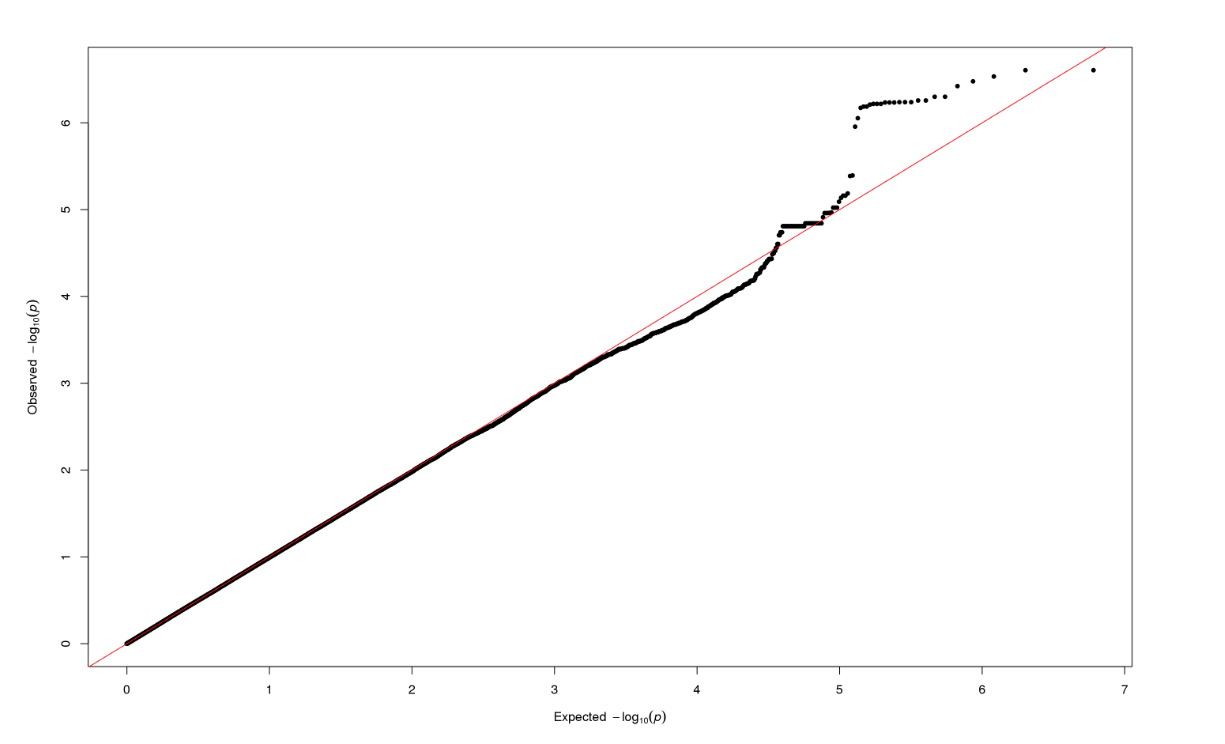

Supplement: Supplementary file 2 — Figure S2. QQ plot of the relationship between expected and observed distribution during weight loss period. Quantile-quantile plot for the analysis of the weight loss period. The relationship between observed (y-axis) and expected (x-axis) distribution. The statistical significance is measured by the negative log of the corresponding p-value for each SNP. (JPEG 94 kb) [file 12263_2018_597_MOESM2_ESM.jpg]
