# Supplementary material for: Analysis of circulating angiopoietin-like protein 3 and genetic variants in lipid metabolism and liver health: the DiOGenes study
Source: Genes Nutr. 2018 Apr 2;13:7. doi: 10.1186/s12263-018-0597-3 (PMC5879874; doi:10.1186/s12263-018-0597-3)
Supplement: Supplementary file 3 — Table S1. Effect of rs4360730 on BMI, Lipid Profile and Liver Markers. Table S2 Effect of rs9994520 on BMI, Lipid Profile and Liver Markers. (DOCX 21 kb) [file 12263_2018_597_MOESM3_ESM.docx]

**Table S1: Effect of rs4360730 on BMI, Lipid Profile and Liver Markers**

| **Baseline** | T/T (n=699) | T/C (n=75) | C/C (n=1) |  |  |
| --- | --- | --- | --- | --- | --- |
|  | Mean ± sd for each genotype | | | β (95%CI) | p |
| AST (RFU)* | 301.4±169.4 | 290.9±174.5 | 210±NA | -20.1 (-45.0;4.79) | 0.078 |
| CK-18 (RFU)* | 220.2±128.7 | 214.8±145.3 | 337±NA | -19.4 (-43.9;5.15) | 0.086 |
| **Weight loss period** | |  |  |  |  |
| AST (RFU) | 0.93±218.3 | 5.42±239.5 | 18.0±NA | 3.71 (-54.3;61.7) | 0.900 |
| CK-18 (RFU) | 1.23±122.9 | -45.1±122.5 | -52.0±NA | -44.8 (-76.9;-12.7) | **0.007** |
| *Data are presented as back-transformed β-coefficients (95%CI) in percent, due to log transformation of the variable before analysis. The regression models were adjusted for center, age, gender and BMI. Models with data from the weight loss period were further adjusted for the change in BMI due to the weight loss. P-value highlighted in bold are significant upon Bonferroni correction. AST, aspartate aminotransferase; CI, confidence interval; CK-18, Cytokeratin 18; RFU, relative fluorescence units; sd, standard deviation; TG, triglycerides. | | | | | |

**Table S2: Effect of rs9994520 on BMI, Lipid Profile and Liver Markers**

| **Baseline** | C/C (n=382) | G/C (n=329) | G/G (n=68) |  |  |
| --- | --- | --- | --- | --- | --- |
|  | Mean ± sd for each genotype | | | β (95%CI) | p |
| AST (RFU)* | 295.8±174.5 | 296.6±168.3 | 317.7±157.4 | 6.58 (-5.77;18.9) | 0.312 |
| CK-18 (RFU)* | 225.0±131.1 | 211.4±128.1 | 240.4±137.9 | -1.54 (-13.6;10.5) | 0.801 |
| **Weight loss period** | |  |  |  |  |
| AST (RFU) | -9.46±234.5 | 8.77±202.9 | 3.77±210.1 | 13.1 (-15.8;42.1) | 0.374 |
| CK-18 (RFU) | -1.02±128.0 | -7.55±110.7 | -5.47±142.0 | -4.30 (-20.3;11.7) | 0.600 |
| *Data are presented as back-transformed β-coefficients (95%CI) in percent, due to log transformation of the variable before analysis. The regression models were adjusted for center, age, gender and BMI. Models with data from the weight loss period were further adjusted for the change in BMI due to the weight loss. AST, aspartate aminotransferase; CI, confidence interval; CK-18, Cytokeratin 18; RFU, relative fluorescence units; sd, standard deviation; TG, triglycerides. | | | | | |
